# From brain drain to brain circulation: evidence from women scientists in OWSD Honduras National Chapter

**DOI:** 10.3389/frma.2026.1861772

**Published:** 2026-07-21

**Authors:** Johana Cabrera-Medina, Indira Ríos, María José Morales Gámez, Dariana Avila-Velasquez, Antonella Rivera, Sindy Vanesa Acosta Rodezno, Larissa Acosta Salgado, Julia Marcela Zelaya, Karina Aquino Valle, Fabiola Rodríguez Vásquez, Karen Orellana-Reyes, Eleana Cano, Isabel Alejandra Cruz, Lariza Sandoval, Melissa Maria Cruz Torres

**Affiliations:** 1 School of Psychology, Faculty of Social Sciences and Humanities, Universidad Autonóma de Chile, Santiago, Chile; 2 Organization for Women in Science for the Developing World—Honduras Chapter, Tegucigalpa, Honduras; 3 Graduate Program in Education, Faculty of Education, Universidade Federal de Goiás, Goiânia, Brazil; 4 Institute of Water and Environmental Engineering, Universitat Politècnica de València, Valencia, Spain; 5 Coral Reef Alliance, Conservation Science, San Francisco, CA, United States; 6 Plataforma da Caracterização Química da Biodiversidade, SENAI Institute of Innovation in Biodiversity and Circular Economy, Brasília, Brazil; 7 Department of Molecular Biology, Galileo University, Guatemala City, Guatemala; 8 Department of Natural Resources and the Environment, Cornell University, Ithaca, NY, United States; 9 Faculty of Chemical Sciences and Pharmacy, National Autonomous University of Honduras, Tegucigalpa, Honduras; 10 Pharmaceutical Research Group, Faculty of Pharmacy, University of Granada, Granada, Spain; 11 School of Physics, Faculty of Science, National Autonomous University of Honduras, Tegucigalpa, Honduras

**Keywords:** academic mobility, brain circulation, brain linkage, Honduras, OWSD, scientific diaspora, scientific networks, women scientists

## Abstract

**Introduction:**

Honduras invests minimally in research and development (0.06% of GDP), contributing to highly qualified professional emigration. When individuals migrate, they may return to Honduras or become part of the scientific diaspora. The exchange of knowledge between professionals abroad and those in their country of origin constitutes “brain circulation”, a process with significant potential to support scientific ecosystems in contexts of low research and development investment. This study examined how the Organization for Women in Science for the Developing World (OWSD) Honduras National Chapter fosters brain circulation and supports scientific development through networks connecting local and diaspora women scientists.

**Methods:**

A sequential explanatory-exploratory mixed-methods design analyzed OWSD Honduras National Chapter membership (*N* = 171, established 2020), with the quantitative phase examining geographic distribution and disciplinary composition, and the qualitative phase employing semi-structured interviews to comprehend migration motivations, professional trajectories, gender barriers, and engagement with Honduras.

**Results:**

Findings showed that Honduran women scientists represented diverse academic disciplines, and their diaspora is found across 13 countries. While structural factors such as limited research infrastructure and funding constraints drive scientist migration, OWSD Honduras National Chapter serves as a space where brain circulation persists and has potential to thrive. Diaspora and returnee scientists engage with this organization to participate in knowledge exchange, access collaborative projects, and develop mentoring relationships. Participants identified OWSD Honduras National Chapter as a safe, non-competitive platform strengthening brain circulation through visibility, networking, and coordination activities, though sustainability and operational capacity challenges were noted.

**Discussion:**

OWSD Honduras National Chapter exemplifies how global entities with local ties can successfully connect diaspora and national scientific communities and contribute to scientific development despite structural constraints. By documenting brain circulation patterns among women scientists in Honduras, this study reveals specific opportunities for system improvements tailored to the local context.

## 1 Introduction

Globally, women constitute a minority within the research community, as only one in three individuals engaged in research is female; that is, they represent just one-third of researchers worldwide (UNESCO, 2024). This gap limits the capacity of scientific systems to respond to global challenges (UNESCO, 2025). Women in scientifically lagging countries face multiple challenges to engage in research and these challenges are compounded by caregiving responsibilities (Quadrio-Curzio et al., 2020). Consequently, mobility between different countries becomes a key pathway for women aspiring to develop scientific careers.

“Brain drain” and “brain circulation” are well established terms used to describe different interpretations of the international mobility of scientific, academic, or highly qualified individuals. In this manuscript, “brain drain” refers to the emigration of highly skilled individuals and the potential loss of knowledge, human capital, or scientific capacity for the country of origin (Carrington and Detragiache, 1999; Docquier and Rapoport, 2012). In contrast, “brain circulation” refers to the transnational circulation of knowledge, skills, networks, and collaborations generated through the mobility and continued engagement of highly qualified individuals, without depending exclusively on their permanent return (Tejada, 2012). While these two terms have different interpretations, they are rooted on the mobility of individuals. Early empirical studies on this process identified the United States and other countries of the Organization for Economic Cooperation and Development (OECD) as destinations for people with tertiary education (Carrington and Detragiache, 1999). In Latin America, since the 1960s, destination countries have included Mexico, Uruguay, Argentina, and Brazil (Pedone and Alfaro, 2018). Subsequent research revealed how Central America and Africa are the regions with high skilled emigration, when excluding small island states and considering countries with populations above four million (Docquier and Rapoport, 2008). The “brain circulation” perspective further develops the mobility concept by describing the communities that maintain links with their countries of origin. These communities and their transnational networks are known as “scientific diasporas”, as they include scientists living or working abroad who maintain actual or potential academic, professional, or collaborative links with their country of origin and may promote knowledge circulation between destination and origin countries (Pellegrino, 2001).^1^ Additionally, it is important to point out that some highly skilled individuals are part of a community of returnees that may also promote circulation of knowledge with the expertise gained during their time abroad.

For developing countries such as Honduras, leveraging the knowledge of its diaspora and returnee scientists could be key to driving scientific and technological advancement. However, Honduras faces major obstacles: national investment in research and development of just 0.06% of GDP (World Bank, 2026) and the absence of official and governmental mechanisms to connect with its diaspora, impeding efforts to channel knowledge and contributions (Bonilla et al., 2022). Returnee scientists may also encounter barriers, re-entry shock and psychological stress, limited local mentorship and networking, professional and career hurdles, academic isolation, and limited research infrastructure and resources (Alsulami, 2021; Kahn and MacGarvie, 2016; Murakami, 2014). Additionally, Honduran women in science face other challenges like fighting against stereotypes and the scarcity or lack of awareness of role models (Ruiz Castro, 2025). In this context, organizations or entities can be spaces for coordination among local women scientists, returnees, and members of the scientific diaspora.

In Honduras, scientific infrastructure and research development are incipient and growing through the participation of universities, research institutes, professional associations, and national scientific organizations. Relevant actors include the Honduran National Academy of Sciences, research institutes affiliated with national universities, and multidisciplinary networks such as Honduras Global (Bonilla et al., 2021, 2022). The latter promotes collaboration among Honduran professionals and scientists both nationally and internationally. UNESCO's Organization of Women for the Developing World (OWSD) is another relevant actor facilitating connections between local women scientists and the diaspora.

Comprising a network of over 12,000 female researchers in emerging economies, OWSD is dedicated to elevating the role of women from the Global South in STEM+ fields. The organization's global operations are divided into four geographic regions, which are overseen by an Executive Board appointed by the General Assembly of its members. Day-to-day administration and program execution are managed by a central Secretariat. At the local level, these efforts are channeled through 57 national chapters, such as the Honduran chapter, which operates under the Latin America and the Caribbean (LAC) regional division (Organization for Women in Science for the Developing World (OWSD), 2010).

The OWSD Honduras National Chapter, founded in 2020 as a grassroots initiative, has created collaboration and knowledge-sharing networks among women scientists inside and outside the country (Avila-Velasquez et al., 2025). To promote the continuity of academic and professional links beyond national borders, OWSD Honduras National Chapter conducts outreach activities, workshops, symposiums, and initiatives that foster engagement within the national research community.

Networks that leverage the scientific diaspora and returnees may operate as intermediary nodes for scientific exchange. Bonilla et al. (2022) studied the Honduran scientific diaspora within the framework of science diplomacy, analyzing perceptions of the term and forms of interaction between diaspora actors and Honduras. Their study treats OWSD Honduras National Chapter as a partial, context-specific case within a broader comparative analysis of Central American scientific communities, focusing on the 2021–2022 period and on the intermediary role of different organizations in scientific engagement (Bonilla et al., 2022). While valuable, this approach does not fully explore OWSD Honduras National Chapter as a women-centered network or systematically characterize its membership and the experiences of its returnees and diaspora members. Focusing on women scientists in the scientific diaspora and on returnees is important, as critical reviews of the literature in Latin America have pointed out that mobility studies have adopted a homogeneous and predominantly male perspective, relegating the analysis of gender and social class and thus limiting the understanding of women's trajectories (Pedone and Alfaro, 2018). Furthermore, focusing in Honduras, is necessary to contribute knowledge for scientific development through available resources and networks like diasporas. For instance, while Avila-Velasquez et al. (2025), documented the emergence and growth of the OWSD Honduras National chapter, they did not examine its role in brain circulation.

Building on this previous research, this study analyzes how OWSD Honduras National Chapter contributes to brain circulation and equitable scientific development in Honduras, grounded in the trajectories, experiences, and perceptions of Honduran women scientists who are currently part of the scientific diaspora, as well as those who have returned to Honduras. The specific objectives were: (1) Characterize the geographic distribution and disciplinary diversity of members of the OWSD Honduras National Chapter who currently belong to the scientific diaspora. (2) Explore the professional trajectories, mobility motivations, gender-related barriers, and perceived scientific opportunities of Honduran scientific diaspora and returnees. (3) Identify recommendations proposed by scientific diaspora and returnees, aimed at strengthening their engagement with Honduran science and promoting equitable scientific development in the country. Findings are expected to provide insights for similar organizations and contexts, informing strategies to enhance brain circulation and foster mutual growth among diaspora women, returnees, and national scientists.

## 2 Methodology

In this study a mixed-methods approach was employed (Creswell, 2005; Ivankova et al., 2006; Tashakkori and Teddlie, 2003). A quantitative phase fulfilled the objective of characterizing the scientific diaspora within the OWSD Honduras National Chapter, and it was followed by a qualitative phase that led to direct engagement with women scientists who are part of the diaspora currently as well as returnees. The qualitative phase led to the characterization of factors that shape women scientists' professional experiences and compiled their recommendations for the strengthening of the OWSD network and Honduran scientific development.

### 2.1 Quantitative phase

Data from the official OWSD website (https://owsd.net/network/honduras) was used to characterize the OWSD Honduras National Chapter members of the scientific diaspora and to generate an empirical basis for the subsequent qualitative inquiry. The OWSD Honduras National Chapter Members database included 171 registered members as of 2025. This registry represents women who, in addition to being Honduran scientists, voluntarily chose to join the network following the outreach and awareness campaigns conducted since its creation in 2020, and who met the organization's membership criteria (Organization for Women in Science for the Developing World (OWSD), 2010). Therefore, a potential source of selection bias should be acknowledged, as the sample may overrepresent women scientists who are already connected to professional or scientific networks, have greater social capital, are more internationally mobile, or have stronger affinity with gender equality and women-in-science agendas. As a result, the quantitative findings, including the proportion of members residing abroad, the number of countries represented, and the disciplinary distribution of the diaspora, should not be interpreted as representative of all Honduran women scientists or of the Honduran scientific diaspora as a whole. Rather, these findings describe the composition of the OWSD Honduras National Chapter membership and provide a context-specific basis for examining how a women-in-science network may foster connectivity, visibility, and brain circulation among its members.

A census approach was applied to include all available data from the OWSD Honduras National Chapter registry. The primary source for the quantitative dataset was the official OWSD database provided to the authors by the OWSD secretariat. Because some variables available in public member profiles were incomplete or not fully included in the official database, publicly available information from the OWSD website was used to complement the dataset where necessary. The final structured dataset included variables such as country/city of residence, type of membership, academic discipline and home institution. Patterns of geographic distribution, disciplinary composition, and international mobility within the network were examined using descriptive statistical summaries, including frequencies and proportions comparing members residing in Honduras and those in the diaspora.

Disciplinary fields were obtained from the official OWSD database and retained using OWSD's original taxonomy. The taxonomy includes 11 disciplinary fields, which OWSD uses to organize fellowships, awards, and member profiles. Data sorting and analyses were conducted using R statistical software (R Core Team, 2025). Descriptive analyses were performed using the “tidyverse” (Wickham et al., 2019), and data visualization was carried out using the “ggplot2” package (Wickham, 2009). Findings from this phase guided the purposive selection of participants for semi-structured interviews in the qualitative phase, through the identification of key profiles: scientific diaspora members, returnees, and nationally based but internationally connected members.

### 2.2 Qualitative phase

An interpretive approach was adopted to gain in-depth understanding of the meanings and experiences Honduran women scientists attribute to their professional trajectories, mobility processes, role within the diaspora or experiences of returning home, and participation in scientific networks. Understanding scientific mobility from women's perspectives, including motivations related to emigration, gender barriers, professional survival strategies, and forms of collaboration (Anastasiadou et al., 2024; González Ramos and Bosch, 2012), requires engaging directly with the women who have experienced these processes. It also calls for an analytical perspective sensitive to the Honduran context in which these trajectories unfold. The qualitative phase involved semi-structured interviews using purposive sampling (Patton, 2015) of the entire registry, informed by the results from the quantitative phase.

The purposive sampling initially included 20 participants (*n* = 20) from the OWSD Honduras National Chapter, with 10 diaspora scientists and 10 returnees found on the official OWSD website. Selection was guided by homogeneity and heterogeneity criteria. Homogeneity criteria ensured coherence within the analytical universe of the study: (1) being a Honduran woman, (2) having membership in OWSD Honduras National Chapter, (3) possessing an academic background, and (4) participating or having prior participation in scientific or academic mobility processes. In turn, heterogeneity criteria ensured diversity across both analytical profiles (diaspora scientist or returnee), and academic discipline and level (master's or doctoral degree). Following the database review and coordination phase, the sample composition was adjusted to include 11 diaspora scientists and nine returnees, reflecting participant availability and willingness to be part of the study. However, one returnee became unresponsive during the scheduling process, resulting in a final sample of *n* = 19, consisting of 11 diaspora scientists (*n* = 11) and eight returnees (*n* = 8) who were interviewed.

In preparation for the semi-structured interviews, all interviewers (i.e., members of the research team) participated in an online training session. The session covered the structure and purpose of the interview, the type of information sought from participants, and strategies for conducting interviews in a consistent and non-directive manner. This process aimed to ensure methodological consistency across interviews conducted by different team members.

Interviews were conducted virtually and lasted approximately 45–60 min. Diaspora scientists (*n* = 11) were distributed across three regions: Latin America (*n* = 5), North America (*n* = 3), and Europe (*n* = 3). Participants held doctoral-level (*n* = 8) and master's-level (*n* = 11) training. The sample was interdisciplinary, spanning six broad scientific domains (i.e., academic disciplines): social and economic sciences, biological systems and life sciences, medical and health sciences including neuroscience, engineering and physical sciences, chemical sciences, and computing and information technology.

After the interviews a thematic analysis involving six stages was conducted to identify patterns and themes related to the objectives of this research (Braun and Clarke, 2006): (1) Data familiarization: a group of researchers transcribed the interviews and engaged in repeated readings to become deeply familiar with the content. (2) Initial code generation: coding was conducted both inductively and deductively, allowing the analytical process to be grounded in the data while remaining theoretically informed. To support the coding process, a deductively developed codebook was constructed based on the theoretical framework and research objectives, specifying the definition and scope of each code to ensure consistency across coders. However, the coding process also allowed for the emergence of new inductively generated codes directly from the data. Each interview was coded independently by two researchers. Discrepancies between coders were resolved through a process of comparison, consensus-building, and thematic adjustment, to deepen the understanding of the phenomenon under study rather than seeking mere agreement. (3) Theme search: codes were grouped into potential themes, and all relevant data extracts were assigned to each theme. With this grouping process transversal patterns across the entire data were identified, thereby establishing relationships across transcripts. (4) Theme review: themes were refined collectively through a process of comparison, consensus-building, and category adjustment, prioritizing a deeper understanding of the phenomenon over simple agreement between researchers. (5) Definition and naming of themes: each theme was defined and named, contributing to the construction of a coherent narrative of the findings. Given the volume and heterogeneity of the themes identified, and the need to respond coherently to the multiple objectives of the study, themes were subsequently grouped into broader analytical axes. Each axis functions as a higher-order organizing category that articulates a set of related themes, facilitating both the interpretation of findings and their structured presentation in relation to the research objectives. (6) Report production: sample extracts were selected to illustrate the themes, and results were synthesized in alignment with the objectives of the study.

Important methodological considerations are recognized in this study: First, data was not collected from an original sampling design tailored to this study, instead the available database from OWSD was used. Second, generalizations cannot be made to the entire Honduran women scientist population, although this study can provide a valuable reference to part of that population. Third, the study is cross-sectional and captures trajectories, identities, and perceptions at a single point in time, preventing longitudinal analysis across professional phases. Fourth, focusing on members of OWSD Honduras National Chapter did not allow for systematic gender comparisons. Despite these limitations, insight was obtained on how women-in-science networks in the context of Honduras may contribute to brain circulation.

## 3 Results

### 3.1 Quantitative results

#### 3.1.1 Geographic distribution of the scientific diaspora

Of the total 171 members of OWSD Honduras National Chapter in 2025, 130 resided in Honduras, and 41 women scientists (24%) resided outside Honduras at the time of analysis and were classified as part of the scientific diaspora. Overall, the diaspora exhibited a broad international dispersion, with members distributed across 13 countries primarily in the Americas and Europe. The largest concentrations were found in Brazil (*n* = 9), the United States (*n* = 7), and Spain (*n* = 6), followed by Mexico (*n* = 4) and France (*n* = 3). Fewer members resided in Canada, Chile, Costa Rica, and the United Kingdom (*n* = 2 each), while Argentina, Germany, the Netherlands, and Norway each hosted one member.

#### 3.1.2 Composition of academic disciplines

**Figure 1:**
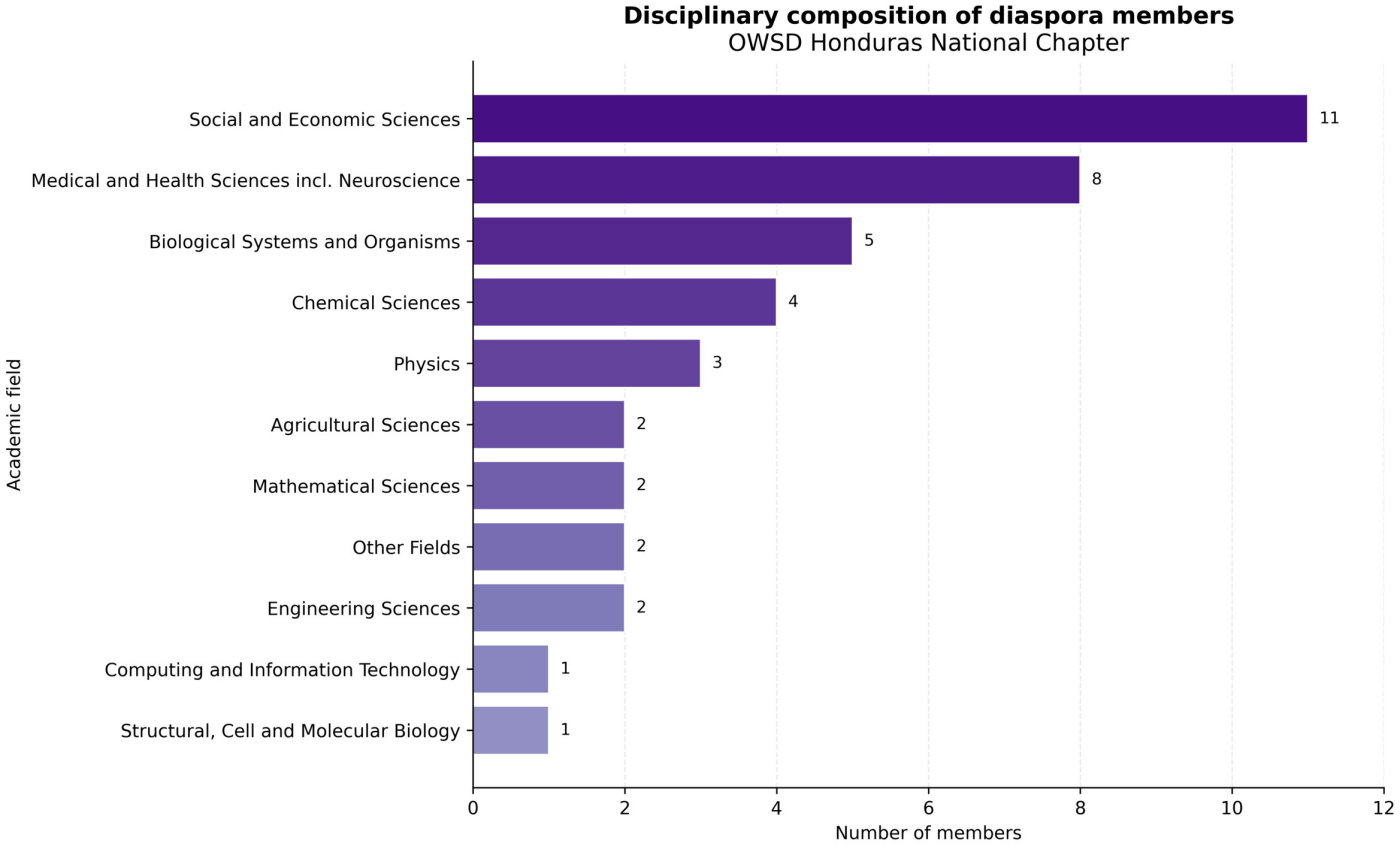
Disciplinary composition of diaspora members of the Honduras National Chapter based on OWSD's own taxonomy.

Diaspora members represented a wide range of academic disciplines across the STEM+ spectrum (Figure 1). The most represented disciplines were Social and Economic Sciences (*n* = 11), Medical and Health Sciences, including Neurosciences (*n* = 8), and Biological Systems and Organisms (*n* = 5). Additional fields included Chemical Sciences (*n* = 4), Physics (*n* = 3), Agricultural Sciences (*n* = 2), Engineering Sciences (*n* = 2), Mathematical Sciences (*n* = 2), Computing and Information Technology (*n* = 1), Structural, Cell, and Molecular Biology (*n* = 1), and other interdisciplinary or unspecified areas (*n* = 2).

### 3.2 Qualitative results

**Figure 2:**
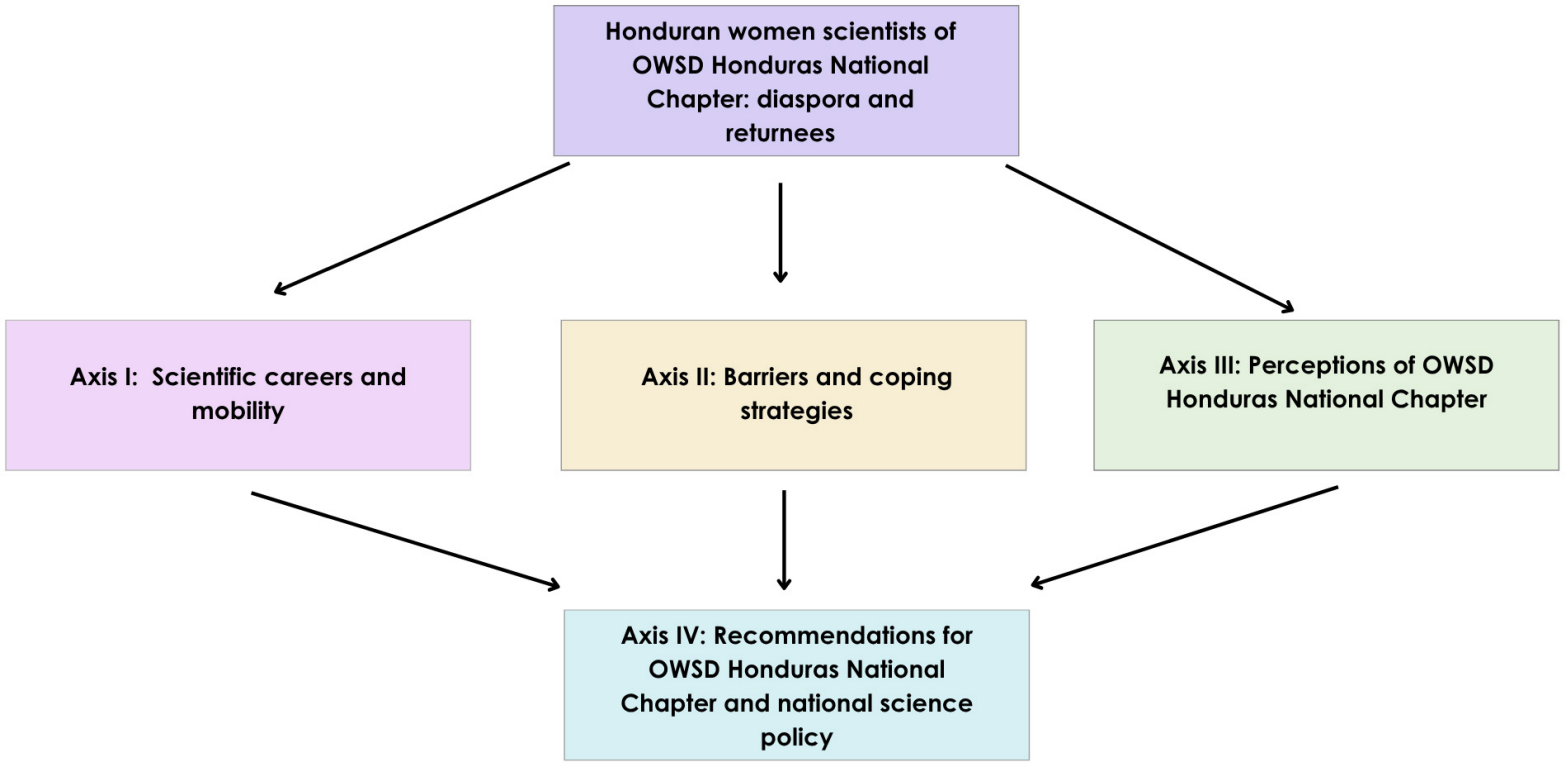
From lived experiences to recommendations: the analytical framework.

**Figure 3:**
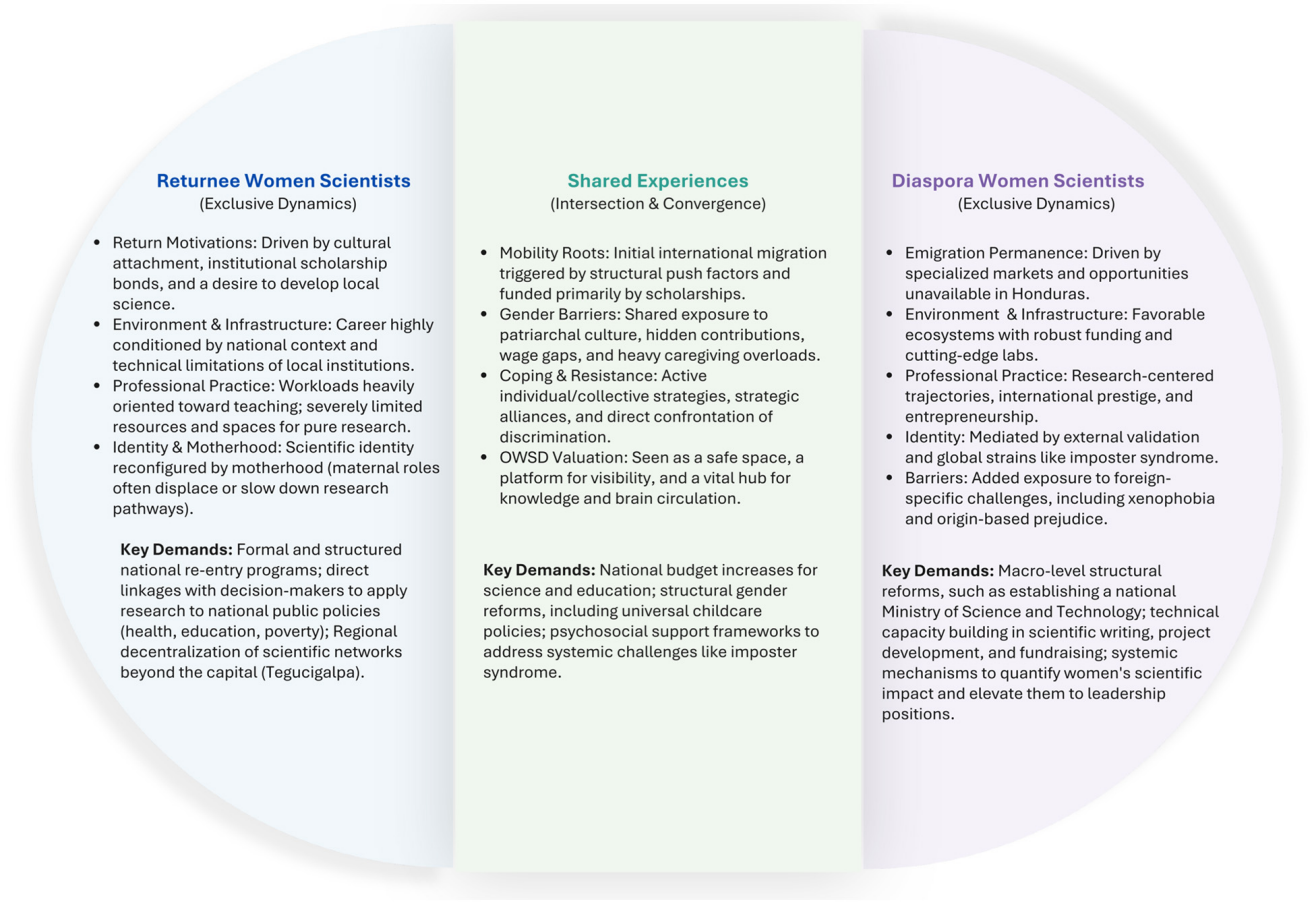
Comparative diagram of qualitative dynamics between returnee and diaspora Honduran women scientists: context-specific trajectories, intersectional barriers, and localized policy demands.

Ten themes were identified by the thematic analysis, belonging to four analytical axes structured in direct correspondence with the specific objectives of the study. The following section presents the in-depth qualitative results, grouped by axis and theme, including codes, definitions, and representative verbatim extracts. An overview of all axes and themes is provided in Table 1, the analytical flow from lived experiences to recommendations in Figure 2, and the key convergences and divergences between returnee and diaspora scientists in Figure 3.

**Table 1:** Summary of results: analytical axes and themes.

Theme number	Analytical axis/theme	Codes	Key findings
T1	Axis 1: scientific careers and mobility Theme: reasons for migration	Structural factors · networks · personal factors	Migration driven primarily by structural factors (economic conditions, insecurity, lack of scientific infrastructure), support networks, and personal motivations.
T2	Axis 1: scientific careers and mobility Theme: scientific identity	Scientific identity · academic life	Dynamic identity with three orientations: disciplinary, social impact, and productivity/teaching. Impostor syndrome present.
T3	Axis 1: scientific careers and mobility Theme: achievements and career trajectories	Scientific recognition · material conditions · career sustainability	High level of qualification in both groups; funding primarily through scholarships.
T4	Axis 2: barriers Theme: patriarchal culture and caregiving responsibilities	Discrimination · structural barriers · emotional and family burdens	Invisibilization, delegitimization, and psychological violence. Caregiving overload with direct impact on academic productivity.
T5	Axis 2: barriers Theme: strategies for overcoming gender barriers	Coping strategies	Individual responses (speaking out, self-care) and collective responses (committees, networks, alliances) to address gender barriers.
T6	Axis 3: perceptions of OWSD Honduras National Chapter Theme: scientific coordination and visibility	Institutional support · visibility	OWSD Honduras National Chapter as a platform for scientific coordination, connection and support among women scientists of different ages and at different stages of their scientific careers, and visibility of their trajectories.
T7	Axis 3: perceptions of OWSD Honduras National Chapter Theme: training and dissemination of opportunities	Training · dissemination	OWSD Honduras National Chapter as a space for disciplinary exchange and dissemination of calls for proposals, funding, and networks.
T8	Axis 3: perceptions of OWSD Honduras National Chapter Theme: structural limitations	Limitations · sustainability	Barriers: time constraints, insufficient internal communication, geographic concentration in the capital of Honduras, lack of stable operational structure.
T9	Axis 4: recommendations Theme: suggestions for OWSD Honduras National Chapter	Institutional strengthening · networks · innovation	Strengthen national and international linkages, establish regional committees, build capacity in fundraising and scientific writing, broaden disciplinary diversity.
T10	Axis 4: recommendations National institutional and policy transformation	Institutional framework · public policies · social impact	Increased science funding, psychosocial support programs, universal childcare policies, institutional ethics frameworks.

#### 3.2.1 Analytical axis I: scientific careers and mobility—(objective 2)

This axis examined how women scientists belonging to the OWSD Honduras National Chapter have experienced mobility throughout their scientific careers. All participants have experienced international migration for educational purposes; that is, they have been part of the scientific diaspora, exemplifying the movement of academic capacities and knowledge. The following themes were identified in this axis:

*Theme 1*. Reasons for migration: structural, personal and network factors

- Codes: “Structural factors, networks, personal factors”
- Theme definition and description: the academic migration of Honduran women scientists arises from a combination of structural factors, support networks, and personal motivations, with structural factors playing a predominant role. Mobility driven by structural factors is primarily associated with the socioeconomic dimension, which reveals unfavorable economic conditions and a context of political instability. Likewise, the social dimension stands out, where insecurity, violence, and the precariousness of basic services such as healthcare act as triggers for emigration or international scientific mobility. The educational dimension highlights the lack of specialized training programs and scientific infrastructure, as well as academic weaknesses, all of which are related to the search for and access to international scholarships.
  Furthermore, structural factors are associated with relational and personal elements in migration such as support networks (academic, professional, friendship-based, and family-based). These support networks promote an emotional environment that provides motivation and leadership role models. Added to these networks are personal and cultural motivations, characterized by a pursuit of self-improvement and personal growth, observed among both diaspora and returnees. Notably, among women scientists in the diaspora, inspiration drawn from other women and the desire for social recognition may be driving forces behind emigration.
- Sample Extract: “*From an early age [...] I had that interest because I saw it at home [...]. My father went abroad to study; in fact, when I was little [...] we were with him [...]. So, I always had that interest, that motivation that once I finished college, I also wanted to go abroad to study” (E19)*

*Theme 2*. Advanced professional trajectories and scientific identity in relation to their training, social contributions, and productivity

- Codes: “Scientific identity, academic life”
- Theme definition and description: scientific identity is defined as a dynamic self-perception that women scientists construct through their trajectories, it is shaped by their careers, relationships, achievements, and personal experiences. Although it is often expressed with confidence and pride, tensions linked to doubts and insecurity may emerge in the diaspora. Scientific identities are diverse in terms of the motivations, interests, and experiences that shape them. Based on this, a classification is proposed that applies to both returnees and those in the diaspora:
  - ° Scientific identity associated with disciplinary training: in this case, learning and development within their academic disciplines form the backbone of their identity.
  - ° Scientific identity oriented toward social impact and contribution: this identity is linked to social contribution. The value of science is defined beyond academic indicators, such as its contribution to society.
  - ° Scientific identity linked to productivity and educational impact: this identity is shaped by the relationship to academic productivity such as projects, publications, and the impact on the education of new generations, especially through teaching and mentoring.
  Within the dynamism of scientific identities, a reconfiguration of scientific identity in relation to motherhood was identified, present only in the case of one returnee, where motherhood displaced scientific work to a secondary position and led to adjustments in her professional trajectory. Scientists in the phase of motherhood were also found in the diaspora; however, no reconfiguration of scientific identity was observed. However, an identity mediated by social recognition and networks was only observed within the diaspora. In this context, scientific identity implies a desire for social validation, and professional trajectories are influenced by the pursuit of both social and academic prestige and recognition.
- Sample extract: “*That imposter syndrome is still there […], that questioning of what it means to be a scientist […]. Especially for me right now because, despite having had a broad career that has allowed me to have different experiences, I still haven't reached certain milestones that, in some way, define what it means to be a researcher [or] a scientist” (E01)*

*Theme 3*. Achievements in the careers of the Honduran women scientific in the scientific diaspora and returnees

- Codes: “Scientific recognition, material conditions for research, sustainability of the scientific career”
- Theme definition and description: the professional trajectories of Honduran women scientists, both returnees and those in the diaspora, reflect international mobility as a strategy for training and seeking opportunities. These trajectories also reflect the circumstances surrounding processes of national reintegration or continued residence abroad. While the predominant source of funding for both groups has been scholarships, in the case of the diaspora, academic loans are also identified as an additional resource.
  As for the returnees, their return is linked to institutional commitments, personal decisions, and a desire to contribute to the country, which generated a sense of attachment. In contrast, among the interviewed scientists in the diaspora, remaining abroad is primarily associated with job opportunities that, according to their own analysis, would be difficult to find in Honduras.
  Both returnees and members of the diaspora have trajectories that include institutional recognition, achievements, and professional development, such as the completion of graduate degrees, entry into teaching and research, participation in outreach activities, and access to funding. Based on these common elements, differences emerge in how professional development materializes depending on the context in which they operate. For returnees, the development of academic life is conditioned by the national context and the characteristics of institutions in Honduras, which limits access to adequate infrastructure and research resources. In this scenario, academic life is predominantly oriented toward teaching, with varying levels of participation in research. In contrast, trajectories of women scientists in the diaspora are characterized by pioneering contributions in their academic work abroad. Some have become founding partners of scientific enterprises, have achieved international prestige, and are recognized as role models in the scientific community. Their infrastructure and working conditions for academic and professional practice are considerably more favorable than those faced by returnees. Furthermore, their trajectories show a strong research component, with access to opportunities and resources that would be difficult to obtain in Honduras. Overall, the careers of both Honduran women returnees and those in the diaspora demonstrate a high level of qualification.
- Sample Extract: “*Perhaps there was an opportunity to return and become a teacher (…) but I liked the research aspect (…) I wanted to continue in the research field (…) perhaps Honduras at that time wasn't developed enough to offer job opportunities in research (…) it's a bit difficult to leave that behind and return (…) to a place with no infrastructure” (E05)*

#### 3.2.2 Analytical axis 2: barriers to a career in science—(objective 2)

This axis gathered experiences that underscore the persistence of gender-based barriers and structural factors that affect scientific careers, both in Honduras and in international contexts. These inequalities manifest themselves in everyday practices, power dynamics, and structural conditions that limit the professional development of women scientists. Nonetheless women have created strategies to overcome these obstacles, the following themes were identified in this axis:

*Theme 4*. Patriarchal culture and caregiving responsibilities in scientific careers

- Codes: “Discrimination, structural obstacles, emotional and family burdens”
- Theme definition and description: this theme is characterized by experiences related to discrimination and gender-based violence in work environments, as well as by the expectation that caregiving responsibilities should fall on women.
  Among returnees and members of the diaspora, experiences revealed patterns of the invisibilization of women scientists' contributions, as well as barriers to their participation in decision-making and in spaces essential for professional growth. Likewise, the accounts revealed a delegitimization of their professional capabilities, aimed at impairing their professional practice. Moreover, psychological forms of sexist aggression were reported, including expectations of a woman's role and her capabilities in academic spaces. All of these obstacles characterize an environment where women scientists are surrounded by stereotypes regarding their capabilities and gender roles. Implications of these everyday dynamics and institutional structures legitimize male power and relegate women to subordinate positions.
  A fundamental aspect that shapes personal and professional life is the overload of caregiving responsibilities. Child-rearing or care for family members who are ill, or have disabilities generates economic, physical, and emotional burdens. Caregiving responsibilities directly impact academic work, as they restrict productivity or reshape professional decisions, potentially impacting the continuity of projects and studies. The result is an ongoing tension in balancing personal life with the demands of an academic career. Although job stability may function as a protective mechanism, difficulties persist in harmonizing personal and professional projects, often leading to the postponement of motherhood or personal life in favor of the scientific career. These challenges are further reinforced by institutional mandates that penalize motherhood and exacerbate the difficulty of reconciling work and personal life.
  The trajectories of women scientists in the diaspora reveal reflections on the contradictions between discourse and practice regarding gender equity. Although academic spaces may publicly promote narratives of equity and the rejection of gender-based violence, in practice, dynamics that reproduce inequalities persist; even colleagues who endorse such discourse may engage in misogynistic behaviors and discriminatory practices. This reflects the deep-rooted patriarchal culture that women scientists confront. In addition, the gender wage gap identified by the interviewees persists across both academic and private sectors (although in academic settings, salary scales have gradually narrowed this gap). Furthermore, the interviews revealed intersectionality in how these experiences can manifest, where sexism, racism, and xenophobia may interact. For xenophobia in particular, these dynamics manifest as prejudice toward their origins, stigmatization of physical differences, and rejection of the foreign.
- Sample Extract: “*The management position (…) is held by a man who always makes comments such as, ‘No, you have to stay at home,' or ‘Because you have children, you can't (…) go on this trip,' (…) often a man's comment is valued more (…) than a woman's contribution” (E19)*

*Theme 5*. Strategies for overcoming gender barriers

- Codes: “Overcoming strategies”
- Theme definition and description: coping strategies in response to gender barriers constitute ways of addressing patriarchal structures. Individual and collective responses were identified among women scientists to confront, resist, and transform barriers. Both returnees and the diaspora use at least one coping strategy, with differences shaped by each academic profile's context.
  At the individual level, actions and attitudes involved the direct confrontation of discourses and practices that question women's professional capabilities. For example, speaking out to denounce violence, challenging unequal structures, and resisting and rejecting the gender wage gap. These actions are complemented by psychosocial self-protection strategies in the face of adverse environments, such as developing self-care practices. Another strategy was expending additional effort to achieve equal institutional support, relevance, and visibility recognition in relation to male peers.
  At the collective level, actions were directed to strengthen professional trajectories, and generate spaces for resistance and mutual support. For example, promoting organizational spaces such as equity committees, women's networks, and creating strategic alliances with key individuals and other women in science. A notable element to highlight in this level, is the identification of the maternal figure, by women scientists, as a reference of strength and perseverance. Finally, an element that appeared only among members of the diaspora was the recognition of the historical struggles of women's movements as part of their own career trajectories, which served them as a tool for challenging inequality in academia.
- Sample Extract: “*There are many things; for example, having to fight (...) to be heard, having to be more direct and having others perceive it as if I'm being rude, but the reality is that they're not listening to me.” (E18)* ^*^The interviewee is referring to academia

#### 3.2.3 Analytical axis 3: perception of the scientific opportunities promoted by OWSD Honduras National Chapter—(objective 2)

This axis identified how women returnees and those who remain in the diaspora generally perceive the OWSD Honduras National Chapter in regard to opportunities. The general perception was that OWSD Honduras National Chapter is a space that opens opportunities for brain circulation and the advancement of their professional development. Participants acknowledged various opportunities but also pointed out limitations that need to be addressed for the chapter's functioning and sustainability. A minority of them stated not receiving support or opportunities from OWSD Honduras National Chapter in their academic careers. The following themes emerged from the data:

*Theme 6*. OWSD Honduras National Chapter as a space for scientific coordination and visibility

- Codes: “Institutional support, relevance and visibility”
- Theme definition and description: OWSD was recognized as a platform and gateway for national and international scientific coordination, collaboration, and contributor to the prestige of scientific careers. OWSD strengthens these connections by enabling the establishment of networks and the articulation of scientific efforts. The chapter was perceived as a space for connection and support among women scientists of different ages and at different stages of their scientific careers, particularly by returnee scientists. OWSD Honduras National Chapter was identified as a platform for enhancing the visibility and positioning of women scientists, whose impact is reflected in their professional trajectories at the local, national, and international levels. It also facilitates scientific dissemination, thus supporting circulation of knowledge in Honduras.
- Sample Extract: “*I love OWSD, how it has evolved, and the opportunity to meet women whose extraordinary work I was not previously aware of.” (E14)*

*Theme 7*. OWSD Honduras National Chapter as a space for scientific training, dissemination of opportunities

- Codes: “Training, dissemination of opportunities”
- Theme definition and description: OWSD was recognized as a key platform for institutional support that connects and supports women scientists across disciplines, strengthening their training and fostering the exchange of knowledge throughout their professional trajectories. From the perspective of some participants, OWSD Honduras National Chapter functions as a space for the dissemination of information on calls for proposals, networks, funding opportunities, and other relevant activities, thereby expanding opportunities for participation and professional development in science.
- Sample Extract: “*For me, OWSD has been like a gateway to information and opportunities that I would not have otherwise known about. It's not just about courses or workshops, but about knowing that there is a network that provides support, shares experiences, and accompanies us, especially when institutional support in Honduras is so limited.” (E.15)*

*Theme 8*. Structural limitations due to existing responsibilities, communication, type of organizational structure and sustainability

- Codes: “Limitations and sustainability”
- Theme definition and description: despite holding generally positive views of the OWSD Honduras National Chapter, returnees and diaspora participants identified limitations that affect their active participation and, consequently, access to the opportunities previously described. Among the main barriers are time constraints and work overload, which hinder sustained involvement due to multiple responsibilities. Similarly, participants highlighted the need for greater interaction and support within OWSD National Honduras Chapter, as some members felt that expectations have not been fully met and that additional spaces for engagement are required, both in person and through internal communication processes. These limitations are reinforced by geographical factors and organizational weaknesses, associated with the absence of a solid operational structure, stable funding, and permanent staff.
- Sample Extract: “*I really value OWSD because it allows me to know that there are other women doing science in Honduras, but sometimes I feel that more interaction is needed. Many activities take place in Tegucigalpa [the capital city of Honduras], and those of us outside the city cannot always participate, so the connection becomes more distant.” (E17)*

#### 3.2.4 Analytical axis 4: recommendations for strengthening ties with the Honduran scientific community and promoting equitable scientific development—(objective 3)

This analytical axis pooled the recommendations formulated by both returnees and those in the diaspora, at various levels. On the one hand, it presents suggestions directed specifically at institutions, including OWSD Honduras National Chapter; on the other hand, it offers country-level recommendations aimed at strengthening women's participation in science and improving international ties. The following themes were identified in the qualitative data:

*Theme 9*. Suggestions specifically directed at OWSD Honduras National Chapter regarding institutional strengthening, advocacy, and innovation

- Codes: “Institutional strengthening and networks, social advocacy, and innovation”
- Theme definition and description: the importance of strengthening international linkages, including the diaspora, was emphasized as a key element for achieving equitable scientific development. The recommendations were to consolidate coordination and articulation strategies among members at the national and international levels, as well as create spaces for interaction that foster networking, disciplinary and multidisciplinary collaboration, and the integration of women scientists within and beyond the network. A proposed action to expand participation and strengthen the organization's institutional presence was the establishment of regional committees or working groups.
  Participants emphasized the need to advance OWSD Honduras National Chapter's sustainability and funding through strategic planning to strengthen the organization, foster participation among members in Honduras and the diaspora, and reinforce its operational structure. Recommendations included capacity building in fundraising, scientific writing, and project development, along with mentoring and cascading training programs. Internal challenges that must be addressed to ensure OWSD Honduras National Chapter's continuity were also identified, such as the need to reflect the disciplinary diversity of women scientists. For example, the social sciences could be brought to the forefront more often. Finally, participants underscored the importance of improving communication and positioning OWSD Honduras National Chapter as a community, political, and scientific actor.
- Sample Extract: “*Strengthen those collaborations a bit more, for example, between the public and private sectors, the academic sector and perhaps private entities, government agencies, or NGOs. Also highlight what the contributions are… how to quantify the impact, right? Of women in publications, in leadership positions, and give them a stronger voice and greater visibility.”—(E05)*

*Theme 10*. Transformation of institutions and strengthening of public policies at the national level

- Codes: “Institutional framework and public policies, innovation and the scientific diaspora, the social impact of science”
- Theme definition and description: Honduran women scientists emphasize the need to strengthen institutions and public policies that promote women's participation and visibility in science. At the institutional level, they proposed psychosocial support programs, including initiatives to address imposter syndrome, increased funding for research aligned with national and gender-related challenges, investment in infrastructure, and the strengthening of science communication directed toward society.
  At the national level, participants highlighted that science funding should respond to the country's priority areas such as health, poverty, education, food security, and employment. They also underscored the importance of increasing the education budget at all levels as a foundation for strengthening the national scientific system, accompanied by ethical frameworks that promote institutional transparency.
  From the perspective of the diaspora, the establishment of a Ministry of Science and Technology was proposed to coordinate research centers and conduct a comprehensive assessment of the state of national science. Returnees, in turn, underscored the importance of re-entry programs and collaborative networks among state, private, and social actors, recognizing that the diaspora's international experience can enhance scientific development and generate a broader societal impact. Finally, participants highlighted the need to adopt best international practices in gender equity, such as the implementation of universal childcare policies, and the coordination of the scientific system through collaborative networks.
- Sample Extract: “*Guide returnees researchers to maintain connections and be able to apply for international projects as well as engage with local public policies in Honduras. Connect decision-makers with women scientists so that we can then work toward solutions for health issues or problems related to what we have come to address.”—(E16)*

To synthesize the important nuances within the sample and directly address the structural variations in the participants' experiences, Figure 3 compares the dynamics between returnee and diaspora women scientists. This analytical framework summarizes their shared institutional barriers and professional values, while explicitly highlighting the context-specific convergences and divergences in their career paths, identity reconfigurations, and structural limitations.

### 3.3 Integrated findings

This subsection reports findings that emerged from the convergence of the quantitative and qualitative phases. Two integrated findings arose from connecting the quantitative characterization of the diaspora (objective 1) with the qualitative analytical axes on scientific careers, mobility, and the perceived role of OWSD Honduras National Chapter (objectives 2 and 3).

**Figure 4:**
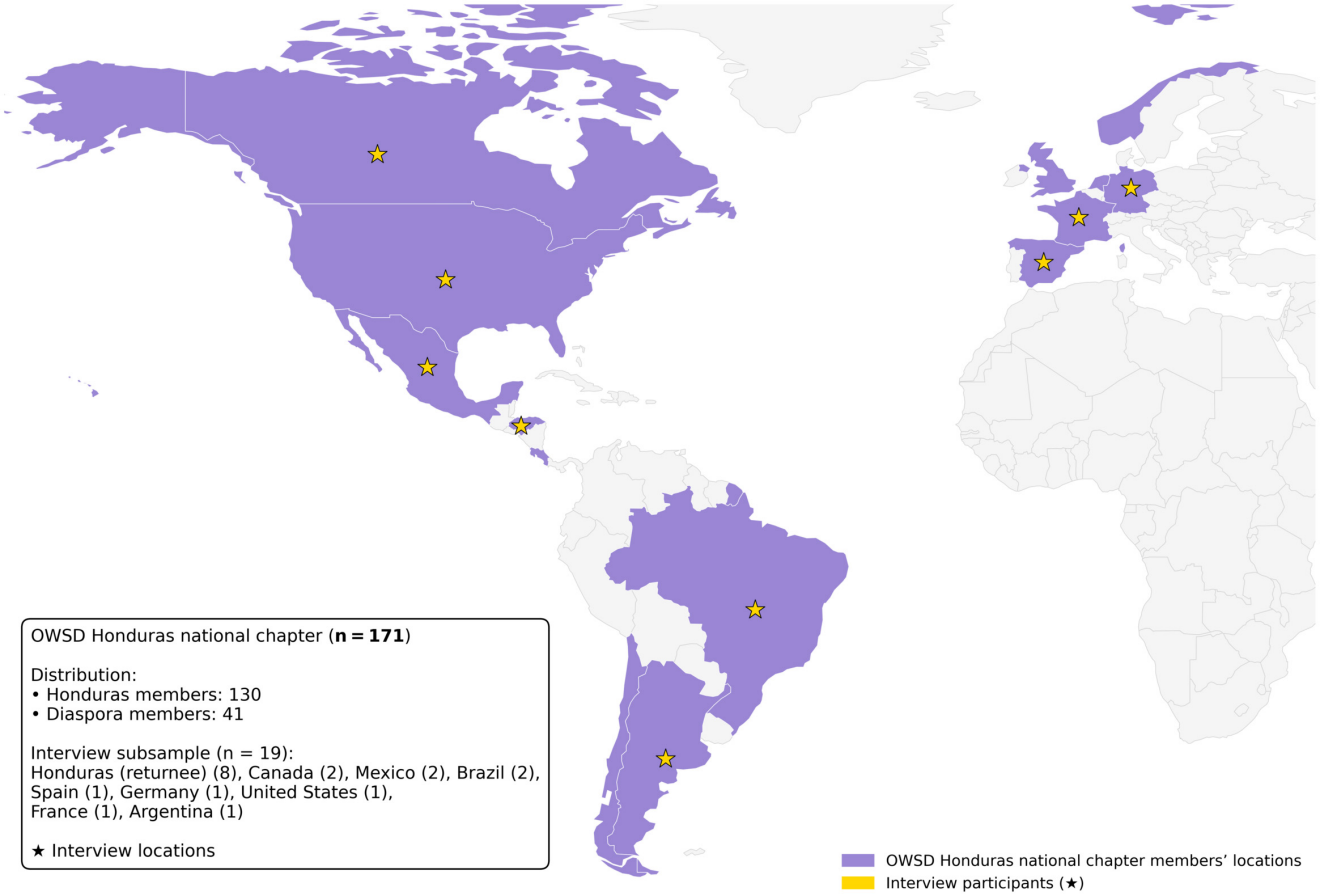
Geographical distribution of OWSD Honduras National Chapter members and interview participants.

First, the mobility of Honduran women scientists operated as structurally conditioned circulation rather than as talent loss. The quantitative phase showed that nearly a quarter of the membership (41 of 171; 24%) was concentrated in three main destinations across distinct regions Brazil, in Latin America; the United States, in North America; and Spain, in Europe which offer greater research investment, infrastructure, and collaboration opportunities than Honduras. These destinations are not equal: the United States and Spain represent established systems in the Global North, whereas Brazil functions as a regional scientific hub within an emerging Latin American context (Figure 4). Integrated with the qualitative accounts of migration and return (Analytical Axis 1), this distribution is explained by structural conditions: participants attributed non-return to employment scarcity and the absence of adequate research infrastructure, while returnees describe their return as driven by institutional commitments, personal reasons, and the desire to contribute to the country. Commitment to Honduras persists across both groups but remains unfulfilled among the diaspora, revealing a gap between willingness and opportunity.

Second, the documented disciplinary diversity reflects potential for knowledge circulation through OWSD Honduras National Chapter. Participants perceived OWSD Honduras National Chapter as a platform for scientific coordination, visibility, training, and the circulation of opportunities (see analytical axis 3), which they also have enabled through activities. The combined evidence indicates that the network sustains active linkages across dispersed locations, enabling members to maintain international ties while contributing to national challenges, thereby reducing fragmentation in knowledge circulation. Taken together, these findings ground a reconceptualization of brain circulation in Honduras as a process of reciprocal scientific engagement in which dispersed women scientists function as active nodes rather than as departed talent.

## 4 Discussion

Brain circulation was evidenced among women scientists in the Honduran scientific ecosystem. This study charts the path of returnee and members of the scientific diaspora belonging to the OWSD Honduras National Chapter, from mobility motivations, trajectories, to their experienced challenges. Ultimately participants' narratives provided insight on how brain circulation can be fostered in Honduras through recommendations for the national science system and for entities such as OWSD Honduras National Chapter.

### 4.1 Mobility of Honduran women scientists

Structural factors encourage the mobility of Honduran women scientists. In this context, migration emerges within a structurally constrained national environment. Honduras faces multiple interconnected challenges including, limited research infrastructure, socioeconomic issues, political instability (BTI, 2026), access to quality basic services (Robayo-Abril et al., 2023), scarce specialized educational programs, gender-based violence and inequality (World Bank, 2023), and the absence of a robust and comprehensive scientific ecosystem. Faced with these limitations, Honduran women seek personal and professional opportunities outside of the country. Although, apart from the influence of structural factors, some women choose migration as an individual decision motivated by aspirations of self-improvement, access to high-quality research environments, and a commitment to contributing to science at both national and international levels. These findings in the present study are consistent with Vega Solís and Gómez Martín (2018), who argued that academic mobility is structurally embedded in a global knowledge economy characterized by asymmetric training capacities and uneven labor markets across countries. For instance, among participants, main destination countries were Brazil, the United States, and Spain, which reflect these asymmetric training capacities between the Honduran scientific ecosystem and these countries' established scientific systems with greater research investment, infrastructure, and international collaboration opportunities, while other destinations showed lower representation, suggesting more dispersed and less consolidated mobility patterns (Barral Morán et al., 2014; Mullet et al., 2017; Sales Oliveira et al., 2025; Santiago et al., 2020).

### 4.2 Returnee and diaspora scientists trajectories

Honduran women scientists in the diaspora and returnees share a common desire to contribute to national research and development despite their varied career trajectories. The participants of this study represented diverse academic disciplines, indicating potential for substantive knowledge exchange and academic success among women despite operating in a country that lags in scientific investment and infrastructure. Several factors motivated their choices to develop academic trajectories as members of the diaspora or returnees. The returnees were driven by institutional commitments arising from scholarship agreements, personal reasons, and genuine aspirations to contribute to the country's development. These motivations have been documented by other case studies (Beoku-Betts 2008; Kahn and MacGarvie, 2016), including Bonilla et al. (2022) conducted in Honduras. Among diaspora members, stated reasons for non-return included employment scarcity and absence of adequate research infrastructure in Honduras, also similarly observed by Beoku-Betts (2008) and Bonilla et al. (2022) in their studies. Although diaspora members develop their careers away from the country of origin, they remain engaged in contributing to Honduras. In fact, returnees and diaspora scientists share a willingness to contribute that is underutilized for brain circulation mechanisms that could leverage both groups' expertise. The motivations to return or remain abroad cannot be separated from obstacles that hinder brain circulation and impact fulfillment in academic careers for these women.

Diaspora members and returnees experience ongoing tensions in the construction of their scientific identities as they face obstacles in Honduras and abroad. Discrimination, harassment, experiences that intersect with factors such as race, country of origin, gender, and inequality in caregiving responsibility, were main obstacles compiled from participant narratives. Such conditions impact academic trajectories of Honduran women by shaping productivity, limiting access to opportunities, and influencing professional decision-making. These findings align with existing literature documenting these dynamics in similar contexts, framing them as structural and intersectional (Beoku-Betts, 2008; Costa et al., 2026; Llorens et al., 2021; Opazo Valenzuela and Pérez-Rincón Fernández, 2023; Márquez Añez and Pastor Gosálbez, 2025; Velicu et al., 2026). The present study offers insights for small scientific systems in low- and lower-middle-income countries, and contributes to the understanding of gender-specific dynamics. To address the underlying causes of these obstacles women face in the Honduran scientific ecosystem, requires comprehensive studies, development of recommendations, application of strategies, and long-term monitoring of outcomes.

### 4.3 Brain circulation: from challenges to recommendations

Brain circulation in Honduras faces structural challenges at national and organizational levels (i.e., within OWSD), yet several recommendations could help it thrive even with these barriers. Participants identified limitations shaped by the country's socioeconomic and institutional context, in which low public investment in science, weak infrastructure for research, and the absence of national policies that support women's scientific careers, create conditions that are structurally inhospitable. All of these challenges were also documented in the national contexts of Honduras, Mexico, and Costa Rica (Bonilla et al., 2022; Gómez-Flores et al., 2022; Jarquín-Solis et al., 2022). Regarding organizational level challenges, specific to OWSD Honduras National Chapter, participants also recognized structural challenges such as the absence of stable funding and permanent staff, insufficient operational structure, and limited capacity for political and community-level advocacy. Nonetheless, recommendations emerged to foster brain circulation.

Promoting an equitable scientific development in Honduras involves countering identified challenges, strengthening women's participation in science and leveraging professional networks. Diaspora members and returnees proposed developing or strengthening policies that promote women's participation, increasing funding for research, and improving or creating research infrastructure to tackle the country's societal priorities. In particular for returnees, including reintegration programs after completing training or a degree abroad was proposed. Additionally, in this study one objective was to identify how engagement of returnees and diaspora members can be strengthened in the country. To that effect, collaborative networks were recommended by participants and we discuss the specific value of OWSD Honduras National Chapter as such a network in the next section. Networks involving the national academics, diaspora, and other societal actors (e.g., non-governmental organizations, government, and private sector) can be spaces of connection to develop research projects (Bonilla et al., 2022; Tejada, 2012).

### 4.4 The value of OWSD Honduras National Chapter for brain circulation

The OWSD Honduras National Chapter serves as a valuable platform where returnees and diaspora members converge to fulfill their desire and commitment to contributing to the Honduran scientific ecosystem. OWSD Honduras National Chapter operationalizes brain circulation by sustaining active linkages among Honduran women scientists in Honduras and abroad. These linkages are enabled by organizational attributes that facilitate these connections. Specifically, both returnees and diaspora scientists find in OWSD a safe, non-competitive environment where they can promote their work and receive recognition for their achievements, research, and expertise. Moreover, belonging to OWSD Honduras National Chapter provides access to a support ecosystem (Márquez Añez and Pastor Gosálbez, 2025) that combats gender biases such as invisibilization, delegitimization, and unequal recognition, which were identified in this study. Consequently, OWSD Honduras National Chapter provides an effective environment for brain circulation as evidenced by this study's findings that also reveal opportunities for growth and improvement. OWSD Honduras National Chapter supports brain circulation through knowledge dissemination activities and establishment of strategic partnerships that connect scientists in Honduras and the diaspora. OWSD Honduras National Chapter operability is supported by Honduras Global, which serves as the chapter's host institution providing access to a broad network of connections (Bonilla et al., 2022). The chapter creates platforms to disseminate members' research such as the National Symposium of Women in Science, held annually since 2020, and the “Hora de Ixmukane” facilitated conservation series, which features scientists based in Honduras and abroad. These two examples reflect circulation of knowledge among members as well as with the general public in Honduras. Initiatives to foster professional exchange between OWSD Honduras National Chapter members and external professionals include establishing connections with organizations and academic networks such as GeoLatinas (a community for Latinas in Earth and Planetary Sciences, https://geolatinas.org), The Conversation (an editorial platform for accessible academic writing), and World Vision Honduras (a non-governmental agency, https://worldvision.hn). Additional activities have included webinars, scientific communication workshops, and mentoring activities.

OWSD Honduras National Chapter' brain circulation efforts are amplified by its member's diverse institutional affiliations which create opportunities for interdisciplinary collaboration and cross-sector linkages among academia, private and public sectors. Thanks to this diversity, members find support for their careers through availability of training opportunities, information about scholarships and research funding, establishment of collaborative projects, access to networking, employment opportunities, and even socioemotional support. Thus, the Honduras chapter fulfills one of OWSD's goals of advancing opportunities for women while also facilitating brain circulation.

Organizations such as OWSD Honduras National Chapter, global in origin but nationally anchored, have potential to maximize brain circulation. Participants identified activities that generate exchange of knowledge or transfer of capacities, such as providing scientific writing training, guidance in grant acquisition, and psychological support for addressing insecurities and impostor syndrome. Additionally, the facilitation of working groups based on academic disciplines could allow OWSD Honduras National Chapter to make advances according to specific disciplinary scopes. The present study has shown that this organization plays a valuable role in a country with limited ecosystem support.

## 5 Conclusion

Women-in-Science networks such as the OWSD Honduras National Chapter create an environment for brain circulation in a country with limited support for the scientific ecosystem. This study documented brain circulation as a pathway from mobility to actions taken and proposed within OWSD Honduras National Chapter. The diaspora and returnees represent the potential for brain circulation through their diverse academic disciplines and rich trajectories. Despite the structural challenges that initially motivated migration and persist today, these women remain connected to Honduras through their membership in the OWSD Honduras National Chapter. Returnees and members of the scientific diaspora share a common desire to engage with the Honduran scientific ecosystem and contribute to knowledge transfer. Evidence of brain circulation within OWSD Honduras National Chapter includes creating platforms for knowledge exchange, establishing strategic partnerships to improve academic opportunities for individuals, and providing peer support for navigating gender-based challenges. Taken together, these findings demonstrate that, in local contexts where diaspora scientists, local academics, and returnees converge within structurally constrained scientific ecosystems, networks are valuable for enhancing knowledge circulation.

## Data Availability

The data supporting the findings of this study are available from the corresponding author upon request. Data will be shared only to the extent that participant confidentiality can be protected.

## Footnotes

1 In this article, whenever the category “*returnee”* is used, it refers to Honduran women scientists who, after completing their studies abroad, voluntarily returned to Honduras. It should **not be confused** with deportations or with forced return as defined by the National Institute of Migration in Honduras, which involves mandatory repatriation processes.
